# Evaluation of autism awareness and knowledge levels among Syrian migrants living in Türkiye

**DOI:** 10.1017/gmh.2024.45

**Published:** 2024-04-01

**Authors:** Selin Davun, Mehmet Akif Sezerol

**Affiliations:** 1 Epidemiology Program, Institute of Health Sciences, Istanbul Medipol University, Istanbul, Türkiye; 2 Department of Public Health, School of Medicine, Istanbul Medipol University, Istanbul, Türkiye; 3 Department of Public Health, School of Pharmacy, Istanbul Medipol University, Istanbul, Türkiye; 4 Sultanbeyli District Health Directorate, Istanbul, Türkiye

**Keywords:** autism, developmental disorders, immigrants, child development

## Abstract

This study was conducted to evaluate the autism knowledge level and awareness of individuals over the age of 18 who applied to immigrant health centers in Istanbul, Gaziantep and Kilis, where the Syrian immigrant population is dense. This cross-sectional study was conducted between December 2022 and April 2023 in 896 immigrants. The sample of the research consists of immigrants residing in Türkiye and who applied to the immigrant health centers in Istanbul, Gaziantep and Kilis for any reason at the time of the research. A questionnaire consisting of three parts was applied to the immigrant people face-to-face. While 38.4% of the participants were female, 61.6% were male. The mean age of the participants is 34.63 ± 10.74. It was determined that people’s place of residence, whether they have children, marital status and income status have significant effects on autism knowledge levels (*p* < 0.001). Since the importance of early diagnosis in autism is known, it is of great importance for people to have knowledge and awareness on this issue. This study will investigate the awareness of the immigrant population, who are faced with traumatic events such as war and migration, and will shed light on future intervention studies.

## Impact statement

Autism is one of the developmental disorders of childhood. The first step in the early diagnosis of autism and its acceptance by the society should be to inform the society about this issue and increase their awareness. There are many studies on autism awareness and knowledge level of the society and some occupational groups. However, there are few studies on immigrant health, which is one of the most important public health problems today. With this study, we emphasized that the awareness of autism, which is a global problem, and the attention of the immigrant population, and the low level of knowledge of this population about autism, and the need for interventions. We compared the factors associated with autism knowledge level and the findings of the immigrant population with other studies conducted in the community. There is also a need for qualitative studies and political regulations on this subject.

## Introduction

Autism was first described by Kanner (1943). It is a developmental disorder with a range of symptoms, including repetitive behaviors and impaired verbal and nonverbal communication (Kanner, 1943). According to the Diagnostic and Statistical Manual of Mental Disorders 3 of the American Psychiatric Association (1989), autism is a common developmental disorder and is distinct from childhood schizophrenia and other psychoses (Le Couteur and Szatmari, 2015).

The prevalence of autism has increased significantly over the past decade, with an estimated prevalence of 1 in 500 reported between 1979 and 2006, and 1 in 161 reported in 2012 (Williams et al., 2006; Elsabbagh et al., 2012). According to the Centers for Disease Control and Prevention, the prevalence of autism in children has risen sharply from 1 in 100 to 2006 to 1 in 88 in 2012, to 1 in 50 in 2013 (Control and Prevention, 2009; Autism and Investigators, 2012; Blumberg et al., 2013). The increasing prevalence of autism is due to multiple factors. These include accuracy of case identification, expansion of diagnostic criteria, increased awareness and knowledge among relevant professionals and the environment and a real increase in incidence (Dillenburger et al., 2013).

Due to the civil war that started in 2011 and continues today, forced migrations were experienced in Syria. Since 2015, Türkiye has been in the position of hosting a large number of refugees, with the majority being Syrians (DANIŞ and Dikmen, 2022). The number of immigrants worldwide is not to be underestimated. According to the most recent figures of the Directorate of Migration Management, on June 22, 2023, there were 3,344,092 registered Syrian individuals under temporary protection status in Türkiye. Approximately one-third of them are children aged 10 and under, born and raised in Türkiye. The efforts of the Child and Rights Protection Platform reveal that 46% of Syrian refugees in Türkiye experience basic issues such as education, 16% suffer from hunger, 7% face housing problems and 7% encounter health issues. For most refugee students, war, deprivation and fear have become the most familiar concepts (SARVAN and Emine, 2020).

There is no epidemiological research on the prevalence of autism among the large mass of immigrants in Türkiye. Four studies have reported on the proportion of immigrants with autism in other countries (Gillberg et al., 1987; Dyches et al., 2004; Kamer et al., 2004). One of these studies showed that there was a higher rate of autism among immigrants compared to nonimmigrants. In addition to the higher prevalence of autism among immigrants, these studies reported genetic disorders, higher rates of brain damage and increased exposure to infectious diseases (Dyches et al., 2004).

In a previous study, the satisfaction rates of parents of children with autism with existing services provided for children with autism in Syria were high. However, the same study found that the parents did not have knowledge about autism or appropriate services and no educational resources in their own language (Mounzer and Alkhteeb, 2009). This lack of knowledge is important because the availability of psychoeducation and services for autism is underdeveloped (Dababnah et al., 2019). Considering the limited research on immigrant populations, there may be a lack of knowledge and awareness, although no definite conclusions can be drawn regarding the prevalence of ASD among immigrants (Bernier, 2021).

Although there are limited studies on the prevalence, awareness and knowledge of autism among immigrants, these families still exist and need health services. A limited understanding of cultural differences can alienate migrant families and lead to low adherence to treatment if interventions are inconsistent with a family’s cultural beliefs. At the beginning of the measures to be taken for this, it is important to understand the knowledge level and awareness of these people on autism.

This study was conducted to evaluate the autism knowledge level and awareness of individuals older than 18 years who applied to immigrant health centers in Istanbul, Gaziantep and Kilis, where the Syrian immigrant population is dense. It will guide the education, prevalence studies and other studies to be carried out for Syrian immigrants in the future.

## Material and methods

### Type of research

This study is a cross-sectional study.

### Study population

This cross-sectional study was conducted between December 2022 and April 2023 in 896 immigrants. The sample of the research consists of immigrants residing in Türkiye and who applied to the immigrant health centers in Istanbul, Gaziantep and Kilis for any reason at the time of the research. The criteria for inclusion in the study were to be over the age of 18, to be a registered immigrant in Türkiye, and to have applied to the immigrant health center for any reason. Immigrant health centers daily 450 considering that the applicant applied, the sample size was calculated as 384 with a 95% confidence interval.

### Measuring tools

A survey consisting of three parts was applied to the immigrant people face-to-face. Before the application of the survey, training was given to the appliers. All interviewers were native speakers of Arabic and administered the survey to the participants in Arabic. In the first part of the survey, sociodemographic questions such as age, sex, education level, marital status, income level and the number of children were included. Income status was evaluated as subjective. In the second part, basic questions about autism were asked, such as whether he had ever heard the word autism before, whether he had any autism in his family or around him, which of the possible causes of autism, and whether he knew which of the symptoms of autism. In the last part, it consists of three likert-type questions about knowledge level, consisting of 33 questions prepared by the researchers by scanning the literature and answered as ‘yes’, ‘no’, ‘I have no idea’. The knowledge level questions were scored as 1 for ‘yes’, 0 for ‘no’ and ‘I have no idea’ answers, and the average score was calculated. According to the average score, if it is below the average, the level of knowledge is interpreted as low, and if it is above the average, the level of knowledge is interpreted as high.

### Statistical analysis

SPSS Program version 22.0 was used for statistical analysis. Continuous variables were expressed as mean ± standard deviation (SD). Categorical variables were expressed as numbers and percentages (%). For statistical analysis of the data, chi-square and Fisher’s exact tests were used to compare the categorical variables between groups. The conformity of the variables to the normal distribution was examined using visual (histogram) and analytical methods (Kolmogorov–Smirnov/Shapiro–Wilk). Logistic regression analysis was performed by dividing the participants into two groups as those below the average and those above the average according to their average scores. Variables that were found to be significant in univariate analyzes were included in the logistic regression analysis. A *p*-value below 0.05 was considered significant statistically.

## Results

Our research was completed with 896 participants. While 38.4% of the participants were female, 61.6% were male. The highest participation in the research was from Istanbul, 62.9% of them. Afterward, Gaziantep with 24.9% and Kilis with 12.2%. The great earthquake that occurred in Türkiye on February 6, 2023 seriously affected the participation rates in Gaziantep and Kilis regions. Then, 81% of the participants are married and 81.3% have at least one child. 35.9% of the participants, constituting the majority, stated that they were primary school graduates. When their income status was questioned, 73.8% of them stated that their income was less than their expenses. When asked if they have ever heard of autism developmental disorder, 34.4% stated that they had not heard of it before. When we asked if there was an individual diagnosed with autism in their family or environment, 86.2% of them answered no. The distribution of sociodemographic characteristics of the participants is shown in Table 1 in detail.

**Table 1.** Distribution of sociodemographic characteristics of participants

n = 896		n	%
Sex	Female	344	38.4%
	Male	552	61.6%
Province	Istanbul	564	62.9%
	Gaziantep	223	24.9%
	Kilis	109	12.2%
Marital status	Married	726	81.0%
	Single	101	11.3%
	Divorced	69	7.7%
Have a child	Yes	728	81.3%
	No	168	18.8%
Literacy	Illiterate	152	16.9%
	Literate	744	83.1%
Education	Primary	402	54.0%
	Secondary	109	14.6%
	High school	96	13.0%
	University	137	18.4%
Income status	Less than expenses	661	73.8%
	Equal to expenses	205	22.9%
	More than expenses	30	3.3%
Has anyone in your family or community been diagnosed with autism?	Yes	124	13.8%
	No	772	86.2%
Have you ever heard of a developmental disorder called autism?	Yes	588	65.6%
	No	308	34.4%
		Mean ± SD	
Age		34.63 ± 10.74	

**Figure 1.**
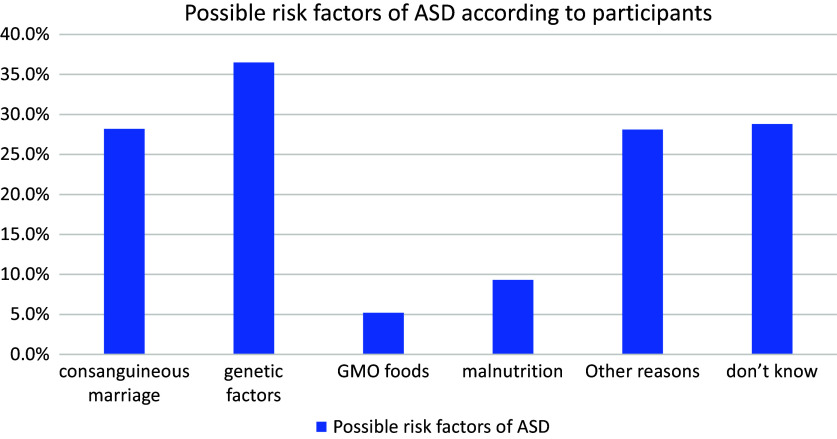
Distribution of reasons of ASD according to participants.

The distribution of the answers given by the participants when asked about the causes of ASD is shown in Figure 1. According to this, the answers were given that genetic factors were effective at most with 36.5%, followed by consanguineous marriage with 28%. Among other answers, GMO foods, malnutrition and other causes were mentioned.

**Figure 2.**
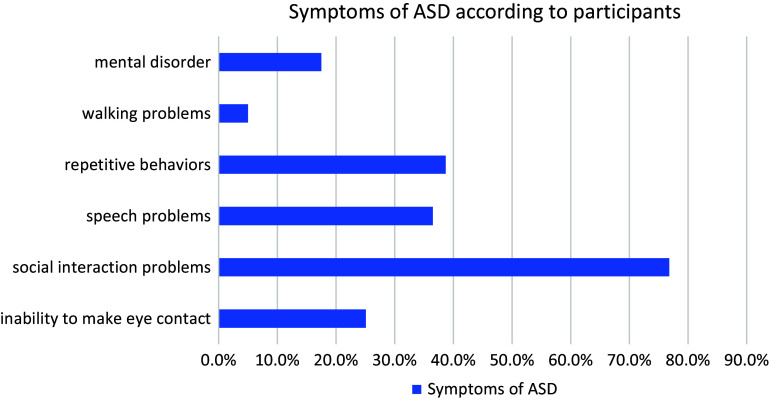
Distribution of possible symptoms of ASD.

When the participants were asked which symptoms of ASD they knew or not, the most; stated that they may experience social communication problems. Afterward, symptoms of repetitive behaviors, speech problems and inability to make eye contact were noted. Then, 5% of the participants stated the symptom of being unable to walk. The distribution of the answers given by the participants is shown in Figure 2.

Participants received one point for correct answers to knowledge questions, and zero point for answers they stated as incorrect or I do not know. Accordingly, the lowest 0 and the highest 33 points can be obtained. In this study, the median value of the scores of the participants was 13.00, with a minimum of 0 and a maximum of 18 points. According to this scoring, those who score below the median value have a low level of knowledge, and those who score above the median value are categorized into two classes. According to this, the difference between the place where the participants lived and their level of knowledge was found to be significant (*p* < 0.001). When the level of knowledge is examined according to whether they have children or not, it has been determined that those who have children show a significantly higher level of knowledge (*p* < 0.001). It was determined that the education and income levels of the participants also had significant effects on their knowledge level (*p* < 0.001). The comparison of autism knowledge levels according to their sociodemographic status is shown in Table 2 in detail.

**Table 2.** Autism knowledge level according to sociodemographic variables

		Level of ASD knowledge				*p*-Value*
		Low		High		
		*n*	%	*n*	%	
Sex	Female	157	45.6	187	54.4	0.125
	Male	281	50.9	271	49.1	
Province	Istanbul	209	37.1	355	62.9	**<0.001**
	Gaziantep	155	69.5	68	30.5	
	Kilis	74	67.9	35	32.1	
Have a child	Yes	335	46.0	393	54.0	**<0.001**
	No	103	61.3	65	38.7	
Literacy	Illiterate	90	10.6	62	7.3	0.523
	Literate	348	41.0	396	46.6	
Education	Primary	123	38.2	199	61.8	**<0.001**
	Secondary	70	64.2	39	35.8	
	High school	43	44.8	53	55.2	
	University	66	50.8	64	49.2	
Marital status	Married	330	45.5	396	54.5	**<0.001**
	Single	73	72.3	28	27.7	
	Divorced	35	50.7	34	49.3	
Has anyone in your family or community been diagnosed with autism?	Yes	64	51.6	60	48.4	0.513
	No	374	48.4	398	51.6	
Have you ever heard of a developmental disorder called autism?	Yes	289	49.1	299	50.9	0.826
	No	149	48.4	159	51.6	
Income status	Less than expenses	293	44.3	368	55.7	**<0.001**
	Equal to expenses	124	60.5	81	39.5	
	More than expenses	21	70.0	9	30.0	

*Note:* Bold values are significant with *p* < 0.05. CI− and CI+ are the lower and upper bounds of the 95% confidence interval. *Chi-square test.

Logistic regression analysis was performed with the variables found to be significant in univariate analyses. According to this, it was determined that the autism knowledge level of the immigrants participating in the research from Gaziantep region was 3.13 times (CI; 2.18–4.51), and the participants from Kilis region had a lower knowledge level of 3.07 times (CI; 1.95–4.84). The analysis results are shown in Table 3 in detail.

**Table 3.** Multivariate analysis of variables^a^

		*p*-Value	OR	95% CI for OR	
				Lower	Upper
Step 1^a^	**Province**	<0.001			
	Gaziantep	<0.001	3.138	2.183	4.512
	Kilis	<0.001	3.079	1.955	4.848
	**Marital status**	0.240			
	Single	0.902	1.034	0.606	1.765
	Divorced	0.160	1.749	0.801	3.816
	**Income status**	0.375			
	Equal to expenses	0.648	0.819	0.349	1.925
	More than expenses	0.898	1.058	0.443	2.528
	**Have a child**	0.592	1.138	0.709	1.829
	**Education**	0.020			
	Primary	0.520	1.203	0.685	2.113
	Secondary	0.147	0.689	0.416	1.139
	High school	0.384	1.312	0.712	2.416
	University	0.719	0.893	0.484	1.651
	**Constant**	0.599	0.747		

a Education status, marital status, income, have a child, Province included to the analysis. Bold values are significant with *p* < 0.05. CI− and CI+ are the lower and upper bonds of the 95% confidence interval.

## Discussion

The quality of life of individuals with ASD and that of their caregivers increase significantly with early diagnosis and intervention. Early diagnosis and intervention are possible by increasing the awareness and knowledge level of society about autism. There are no studies on the knowledge level of autism among Syrian immigrants or the prevalence of autism in this minority population in Türkiye. Although Syrian immigrants are classified as a minority population, there are currently over 3 million Syrian immigrants living in Türkiye, according to data from the Directorate of Migration Management. Each individual with autism who is missed in terms of early diagnosis will create a significant burden both individually and socially in the future. This study, which was conducted to evaluate the knowledge level and awareness of Syrian immigrants of autism, aimed to reveal inspiring findings in the planning of awareness-raising activities for these individuals.

This study was carried out in three provinces (Istanbul, Gaziantep and Kilis), with the highest number of participants included in the study from Istanbul. A major earthquake that occurred on February 6, 2023 in Türkiye coincided with the time during which the study was conducted. The earthquake caused destruction in Gaziantep and Kilis and was one of the main reasons for the lower participation in these regions. In this study, the autism knowledge level of immigrants living in Istanbul was significantly higher than those of immigrants living in Gaziantep and Kilis, with 65.6% of the participants stating that they had heard the word autism. In a study on parents in Türkiye, 92.7% said they had heard the word autism (Can et al., 2021). In a study in Pakistan, 75.2% of participants had heard of autism before (Anwar et al., 2018). In a study in Ireland on 1,205 members of the general population, 82% stated that they had heard of autism (Dillenburger et al., 2013). The participants gave the answer that genetic factors were the most effective for autism symptoms. Similar to this study, in a study on pharmacists in Palestine, 61.4% of the participants stated that genetic factors were the cause of autism (Shawahna et al., 2017). In a study in China, most of the participants stated that inappropriate family education was the cause of autism, and the second was abnormal brain development (Wei et al., 2022).

In general, the median value of autism knowledge level was low among the Syrian immigrants. The knowledge levels of autism of participants who had children were significantly higher than those of participants who did not have children. This finding is in accordance with that of a study conducted in Zambia, where participants who had children had higher levels of awareness and knowledge (Chansa-Kabali et al., 2019). This may be because those who have children show special interest in the characteristics of different conditions and treatment options. Parents may also have obtained some information from interactions with different materials, including prenatal and postnatal visits to family health centers and hospitals.

Although the education level of the participants was quite low, the most reported education level was being primary school graduate. After all, it was illiteracy. In this case, it is expected that the level of knowledge is low, and the relationship was found to be significant according to the level of education. In a study conducted in the general population in the United States, the autism knowledge level was found to be significantly higher in those with a high school or higher education level (Holt and Christensen, 2013). This is an expected situation since those with higher education levels will have easier access to information sources and easier to understand.

Most of the Syrian immigrants participating in this study are married. Autism knowledge levels were found to be significantly higher in those whose marital status was married than those who were single or divorced. In a study conducted with healthcare professionals in Iran, the marital status of married people is higher than those of single people with autism knowledge level (Effatpanah et al., 2019). In another study conducted in Ghana, no significant effect of marital status on autism knowledge was found (Sampson and Sandra, 2018).

In this study, no significant relationship was found between whether or not there was a person diagnosed with autism in their families and relatives and their level of knowledge about autism spectrum disorder. Similarly, in a study conducted in Türkiye, it was determined that those who have relatives with autism do not show completely correct approaches about autism (Arslan, 2023). In the study of Töret et al., the knowledge level of those in their close circle with individuals diagnosed with autism was significantly higher (Töret et al., 2014).

This research was carried out in order to determine the knowledge and awareness of Syrian immigrants about autism and to guide intervention studies on this issue. The research has some limitations and strengths. First of all, the research could not show an equal distribution in all three regions, and there was more participation in Istanbul. The biggest reason for this is the earthquake that took place in Gaziantep, Kilis region in Türkiye during the date of the research and caused destruction. Second, the autism knowledge levels of the participants were measured subjectively, and a scale with validity and reliability was not used.

The research is a strong study with the participation rate and the fact that it was conducted on Syrian immigrants living in Türkiye. In addition, the fact that the answers are made online and by people who know Arabic one-to-one is another strength.

## Conclusion

Research on autism awareness over the past decade shows impressive progress in this regard. More research is needed at the community level to increase autism awareness. This study was conducted to determine the autism awareness and knowledge level of Syrian immigrants. The results of the study showed that there is low knowledge about autism among Syrian immigrants when compared to other social studies. The low level of knowledge and awareness of Syrian immigrants may cause them to be late in a possible situation that may occur in their children. This study will contribute both to interventions to improve the current situation and to early diagnosis of children.

## Data Availability

The data that support the findings of this study are available from the corresponding author.

## References

[r1] Anwar MS, Tahir M, Nusrat K, Khan MR and Khan M (2018) Knowledge, awareness, and perceptions regarding autism among parents in Karachi, Pakistan. Cureus 10(9), e3299. 30443469 10.7759/cureus.3299 PMC6235645

[r2] Arslan K (2023) Otizmli Bireylere Yönelik Toplumsal Farkındalık Düzeyinin İncelenmesi. Avrasya Uluslararası Araştırmalar Dergisi 11(34), 28–47.

[r3] Autism and Investigators DDMNSYP (2012) Prevalence of autism spectrum disorders—Autism and developmental disabilities monitoring network, 14 sites, United States, 2008. Morbidity and Mortality Weekly Report: Surveillance Summaries 61(3), 1–19. 22456193

[r4] Bernier AS (2021) Autism in the context of humanitarian emergency: The lived experiences of Syrian refugee parents of children on the autism Spectrum. Autism 2021, 08–26.

[r5] Blumberg SJ, Bramlett MD, Kogan MD, Schieve LA, Jones JR and Lu MC (2013) *Changes in Prevalence of Parent-Reported Autism Spectrum Disorder in School-Aged US Children: 2007 to 2011–2012.* US Department of Health and Human Services, Centers for Disease Control. 24988818

[r6] Can A, Güner ÇZ, Lüleci E, Bağrıaçık F, Karavuş M, Arı N, Hıdıroğlu S and Altaş ZM (2021) Altı yaş altı çocukların ebeveynlerinde otizm bilgi düzeyinin ölçülmesi. Journal of Turkish Family Physician 12(1), 12–21.

[r7] Chansa-Kabali T, Nyoni J and Mwanza H (2019) Awareness and knowledge associated with autism spectrum disorders among university students in Zambia. Journal of Autism and Developmental Disorders 49, 3571–3581. 31140012 10.1007/s10803-019-04044-7

[r8] Control CfD and Prevention (2009) Prevalence of autism spectrum disorders-autism and developmental disabilities monitoring network, United States, 2006. Morbidity and mortality weekly report. Surveillance summaries (Washington, DC: 2002) 58(10), 1–20. 20023608

[r9] Dababnah S, Habayeb S, Bear BJ and Hussein D (2019) Feasibility of a trauma-informed parent–teacher cooperative training program for Syrian refugee children with autism. Autism 23(5), 1300–1310. 30409031 10.1177/1362361318805368

[r10] DANIŞ D and Dikmen H (2022) Türkiye’de göçmen ve mülteci entegrasyonu: Politikalar, uygulamalar ve zorluklar. İstanbul Ticaret Üniversitesi Sosyal Bilimler Dergisi 21(Özel Sayı), 24–45.

[r11] Dillenburger K, Jordan JA, McKerr L, Devine P and Keenan M (2013) Awareness and knowledge of autism and autism interventions: A general population survey. Research in Autism Spectrum Disorders 7(12), 1558–1567.

[r12] Dyches TT, Wilder LK, Sudweeks RR, Obiakor FE and Algozzine B (2004) Multicultural issues in autism. Journal of Autism and Developmental Disorders 34, 211–222. 15162939 10.1023/b:jadd.0000022611.80478.73

[r13] Effatpanah M, Shariatpanahi G, Sharifi A, Ramaghi R and Tavakolizadeh R (2019) A preliminary survey of autism knowledge and attitude among health care workers and pediatricians in Tehran, Iran. Iranian journal of child neurology 13(2), 29. 31037075 PMC6451856

[r14] Elsabbagh M, Divan G, Koh YJ, Kim YS, Kauchali S, Marcín C, Montiel-Nava C, Patel V, Paula CS, Wang C, Yasamy MT and Fombonne E (2012) Global prevalence of autism and other pervasive developmental disorders. Autism Research 5(3), 160–179. 22495912 10.1002/aur.239 PMC3763210

[r15] Gillberg C, Steffenburg S, Börjesson B and Andersson L (1987) Infantile autism in children of immigrant parents: A population-based study from Göteborg, Sweden. British Journal of Psychiatry 150(6), 856–858. 10.1192/bjp.150.6.856 3651741

[r16] Holt JM and Christensen KM (2013) Utahns’ understanding of autism spectrum disorder. Disability and Health Journal 6(1), 52–62. 23260611 10.1016/j.dhjo.2012.08.002

[r17] Kamer A, Zohar AH, Youngmann R, Diamond GW, Inbar D and Senecky Y (2004) A prevalence estimate of pervasive developmental disorder among immigrants to Israel and Israeli natives: A file review study. Social Psychiatry and Psychiatric Epidemiology 39, 141–145. 15052396 10.1007/s00127-004-0696-x

[r18] Kanner L (1943) Autistic disturbances of affective contact. Nervous child 2(3), 217–250. 4880460

[r19] Le Couteur A and Szatmari P (2015) Autism spectrum disorder. In Thapar A, Pine DS, Leckman JF, Scott S, Snowling MJ and Taylor E (eds), Rutter’s Child and Adolescent Psychiatry. John Wiley & Sons, Ltd. pp. 661–682.

[r20] Mounzer W and Alkhteeb J (2009) Parents’ satisfaction with services provided to their children with autism in Syria. Arabian Academy for Special Education Journal 9(2), 89–107.

[r21] Sampson W-G and Sandra AE (2018) Comparative study on knowledge about autism spectrum disorder among paediatric and psychiatric nurses in public hospitals in Kumasi, Ghana. Clinical practice and epidemiology in mental health: CP & EMH 14, 99. 29785200 10.2174/1745017901814010099 PMC5897989

[r22] Sarvan S and Emine E (2020) MÜLTECİ ÇOCUKLAR ve SORUNLARI. Balıkesir Sağlık Bilimleri Dergisi 9(1), 55–62.

[r23] Shawahna R, Fahed B, Qadri D, Sharawi L, Soroghli M and Dweik M (2017) Awareness and knowledge of autism spectrum disorders among pharmacists: A cross-sectional study in Palestinian pharmacy practice. Journal of Autism and Developmental Disorders 47, 1618–1627. 28251394 10.1007/s10803-017-3085-5

[r24] Töret G, Özdemir S, Selimoğlu ÖG and Özkubat U (2014) Otizmli çocuğa sahip olan ebeveynlerin görüşleri: Otizm tanımlamaları ve otizmin nedenleri. Ankara Üniversitesi Eğitim Bilimleri Fakültesi Özel Eğitim Dergisi 15(01), 1–17.

[r25] Wei H, Li Y, Zhang Y, Luo J, Wang S, Dong Q, Tao Y, Gong L, Feng Y, Shi M, Cao Z, Liu Y, Chen L, Liu X, Dai Y, Qu L, Song Z, Chen J, Li T and Cheng Q (2022) Awareness and knowledge of autism spectrum disorder in Western China: Promoting early identification and intervention. Frontiers in Psychiatry 13, 970611. 36440386 10.3389/fpsyt.2022.970611 PMC9686393

[r26] Williams JG, Higgins JPT and Brayne CEG (2006) Systematic review of prevalence studies of autism spectrum disorders. Archives of Disease in Childhood 91(1), 8–15. 15863467 10.1136/adc.2004.062083 PMC2083083

