# Supplementary material for: Evaluation of autism awareness and knowledge levels among Syrian migrants living in Türkiye
Source: Glob Ment Health (Camb). 2024 Apr 1;11:e44. doi: 10.1017/gmh.2024.45 (PMC11058514; doi:10.1017/gmh.2024.45)
Supplement: Peer review history

# Editor report: Recommendation: Evaluation of autism awareness and knowledge levels among Syrian migrants living in Türkiye — R0/PR2

**DOI:** 10.1017/gmh.2024.45.pr2

**Affiliations:** Department of Global Health and Social Medicine, Harvard Medical School, United States

No accompanying comment.

# Editor report: Decision: Evaluation of autism awareness and knowledge levels among Syrian migrants living in Türkiye — R0/PR3

**DOI:** 10.1017/gmh.2024.45.pr3

**Affiliations:** London School of Hygiene & Tropical Medicine, United Kingdom of Great Britain and Northern Ireland

No accompanying comment.

# Editor report: Recommendation: Evaluation of autism awareness and knowledge levels among Syrian migrants living in Türkiye — R1/PR5

**DOI:** 10.1017/gmh.2024.45.pr5

**Affiliations:** Department of Global Health and Social Medicine, Harvard Medical School, United States

No accompanying comment.

# Editor report: Decision: Evaluation of autism awareness and knowledge levels among Syrian migrants living in Türkiye — R1/PR6

**DOI:** 10.1017/gmh.2024.45.pr6

**Affiliations:** London School of Hygiene & Tropical Medicine, United Kingdom of Great Britain and Northern Ireland

No accompanying comment.

# Editor report: Recommendation: Evaluation of autism awareness and knowledge levels among Syrian migrants living in Türkiye — R2/PR8

**DOI:** 10.1017/gmh.2024.45.pr8

**Affiliations:** Department of Global Health and Social Medicine, Harvard Medical School, United States

No accompanying comment.

# Editor report: Decision: Evaluation of autism awareness and knowledge levels among Syrian migrants living in Türkiye — R2/PR9

**DOI:** 10.1017/gmh.2024.45.pr9

**Affiliations:** London School of Hygiene & Tropical Medicine, United Kingdom of Great Britain and Northern Ireland

No accompanying comment.
